# Comparative Pharmacokinetics Study of Icariin and Icariside II in Rats

**DOI:** 10.3390/molecules201219763

**Published:** 2015-12-01

**Authors:** Tao Cheng, Yong Zhang, Tong Zhang, Lu Lu, Yue Ding, Yuan Zhao

**Affiliations:** 1 School of Pharmacy, Shanghai University of Traditional Chinese Medicine, Shanghai 201203, China; chengtaohx@126.com (T.C.); yjll66@outlook.com (L.L.); 2 Experiment Center for Teaching and Learning, Shanghai University of Traditional Chinese Medicine, Shanghai 201203, China; yongzhang11@163.com (Y.Z.); cmx_1@126.com (Y.Z.)

**Keywords:** icariin, icariside II, UPLC-MS/MS, pharmacokinetic study

## Abstract

To explore the pharmacokinetic properties of icariin (ICA) and icariside II (ICA II) following intragastric and intravenous administration in rats, a rapid and sensitive method by using ultra-performance liquid chromatography–tandem mass spectroscopy (UPLC-MS/MS) was developed and validated for the simultaneous quantification of ICA and ICA II in rat plasma. The quantification was performed by using multiple reaction monitoring of the transitions *m*/*z* 677.1/531.1 for ICA, 515.1/369.1 for ICA II and 463.1/301.1 for diosmetin-7-*O*-β-d-glucopyranoside (IS). The assay showed linearity over the concentration range of 1.03–1032 ng/mL, with correlation coefficients of 0.9983 and 0.9977. Intra- and inter-day precision and accuracy were within 15%. The lower limit of quantification for both ICA and ICA II was 1.03 ng/mL, respectively. The recovery of ICA and ICA II was more than 86.2%. The LC-MS/MS method has been successfully used in the pharmacokinetic studies of ICA and ICA II in rats. The results indicated that 91.2% of ICA was transformed into ICA II after oral administration by rats, whereas only 0.4% of ICA was transformed into ICA II after intravenous administration. A comparison of the pharmacokinetics of ICA and ICA II after oral administration revealed that the *C*_max_ and AUC_0–t_ of ICA II were 3.8 and 13.0 times higher, respectively, than those of ICA. However, after intravenous administration, the *C*_max_ and AUC_0–t_ of ICA II were about only 12.1% and 4.2% of those of ICA. These results suggest that ICA and ICA II have distinct pharmacokinetic properties, and the insights obtained facilitate future pharmacological action studies.

## 1. Introduction

Epimedii folium (EF) is the dried leaves from *Epimedium brevicornu* Maxim., *E. sagittatum* (Sieb.et Zucc.) Maxim., *E. pubescens* Maxim., *E. koreanum* Nakai. EF, also named barrenwort, Yinyanghuo, faeries spleen and copper wire grass, and has been used for thousands of years as a tonic herb for nourishing the kidneys and strengthening the bones in East Asia, particularly in Korea, China and Japan [1]. EF is widely used in clinical settings for its antiosteoporotic activity [2]. Other pharmacological effects of EF, its extracts and active components include improving bone health and cardiovascular function, regulating hormone level, modulating immunological function, enhancing sexual function, and inhibiting tumor growth [3]. Modern clinical pharmacology studies have also demonstrated the potency of EF in treating rheumatic and immune diseases [4,5], hyperglycemia, diabetic complications [6,7,8], and cardiovascular diseases [9]. Icariin (ICA), the most abundant constituent in EF, has been recognized as a unique chemical marker for the quality control of EF in the Chinese Pharmacopoeia [10]. ICA has potential effects against osteoporosis, inflammation, depression, oxidation, atherosclerosis, cancer and insulin resistance [11,12,13,14,15]. However, *in vivo* and *in vitro* metabolism studies have indicated ICA can be transformed into icariside II (ICA II, baohuoside I) by the hydrolysis effects of intestinal microflora. As the *in vivo* predominant bioactive form of ICA, ICA II exerts strong biological activities against inflammation, osteoporosis, hypoxia and cancer, and improves erectile dysfunction [16,17,18,19,20]. Thus, ICA II has attracted increasing research interest.

The process of preparing ICA II from ICA through the enzymatic hydrolysis method, which can yield sufficient amounts of ICA II for use in pharmacological studies, has been developed. In addition, the pharmacodynamic actions differ with the small dissimilarities in the structure of ICA and ICA II. Liu [21] reported that ICA II regulates endothelial cell function by increasing the endothelial nitric oxide synthase (eNOS) expression through the activation of the epidermal growth factor (EGF)-EGFR receptor pathway in porcine aortic endothelial cells. In addition, ICA II enhances osteoblast activity and suppresses osteoclast activity more effectively than ICA does [22], which is consistent with Zhang’s finding that the C-7 hydroxyl group in the structure of ICA II might play a crucial role in the antiosteoclastic activity [23]. Moreover, in a study on drug–drug interaction potential, ICA potently inhibited UGT1A3, whereas ICA II potently inhibited UGT1A4, UGT1A7, UGT1A9, and UGT2B7; this inhibitory activity induces different effects on the metabolism of several endogenous and exogenous substrates. However, most of these studies were conducted *in vitro*, where the *in vivo* environment cannot be completely replicated. The relationship between the structure and physiological processes of ICA and those of ICA II has not been systematically studied using animal experiments. Elucidation of the pharmacokinetic characteristics of ICA and ICA II would clarify the underlying mechanisms of action and help to predict their efficacies.

Pharmacokinetic studies have mostly focused on developing suitable analytical methods for quantifying ICA and ICA II in biological samples. The methods developed using capillary zone electrophoresis [24] and high-performance liquid chromatography (HPLC) [25] can only be applied for quantifying ICA and ICA II in herbs and their extracts because of low sensitivity. Several high-performance LC-tandem mass spectrometry (HPLC-MS/MS) methods have been developed for detecting and quantifying ICA and its metabolites. A rapid and sensitive method to separate and quantify ICA, icariside I, ICA II, icaritin (ICT), and desmethylicaritin in rat serum was developed using LC-MS/MS; this method has been applied to quantify the aforementioned compounds in rat serum after orally administering an Epimedium preparation [26]. Another ultra-performance LC-MS/MS (UPLC-MS/MS) method was applied to detect seven main flavonoids, including ICA and ICA II, in dog plasma and in their pharmacokinetic studies after orally administering a Herba Epimedii extract [27]. To the best of our knowledge, no reports on the application of these methods to the pharmacokinetic study of ICA and ICA II after their intragastric and intravenous administration exist in the literature. In the present study, we developed and validated a UPLC-MS/MS method for the simultaneous determination of ICA and ICA II in rat plasma. This method is adequately sensitive and can be applied in comprehensive pharmacokinetic studies of ICA and ICA II and to elucidate the mechanisms underlying the different efficacies of ICA and ICA II.

## 2. Results and Discussion

### 2.1. Optimization of UPLC-MS/MS Conditions

**Figure 1:**
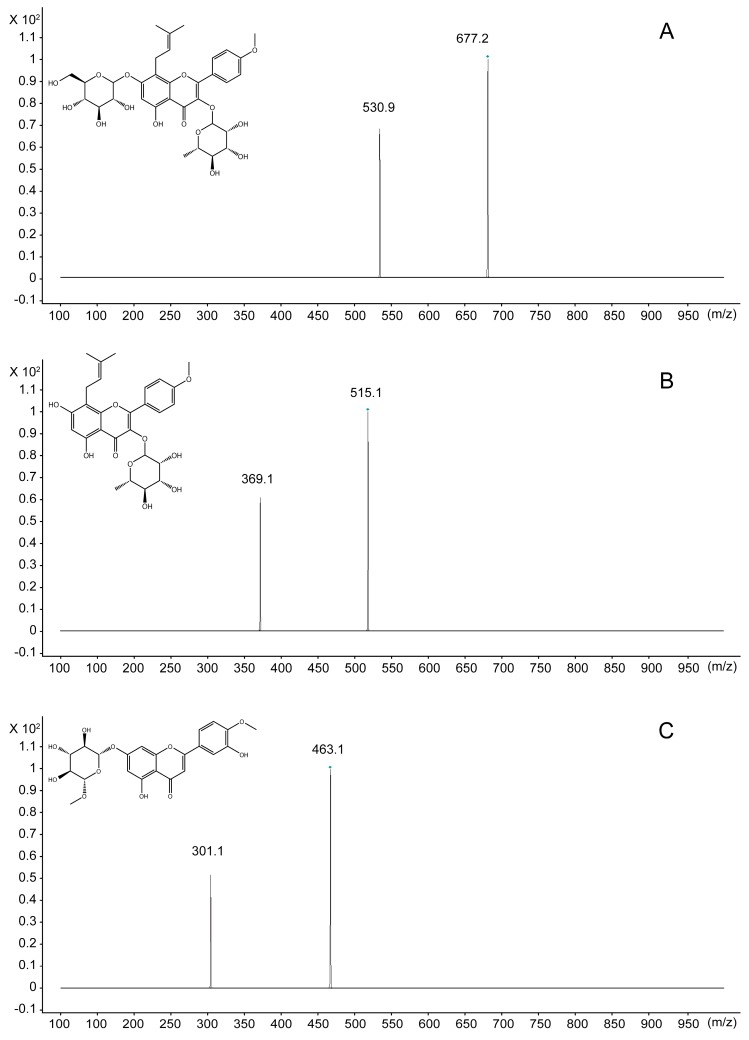
Mass spectra of ICA, ICA II and IS. (**A**) ICA; (**B**) ICA II; (**C**) IS.

For detecting ICA and ICA II in rat plasma and investigating their pharmacokinetics, a UPLC-MS/MS method was developed and validated. To increase the sensitivity of ion response and obtain the baseline separation of ICA and ICA II, chromatographic conditions and MS parameters were optimized. MS determination of ICA, ICA II, and diosmetin-7-*O*-β-d-glucopyranoside (IS) standard solutions in both positive and negative ionization modes was attempted during method development. In the full-scan mode, electrospray ionization (ESI) source in the positive mode had a higher quality resolution and higher intensity to permit quantitative measurement than did the negative mode for both ICA and ICA II. To ascertain the precursor ions and determine product ions for use, the product ions and multiple reaction monitoring (MRM) mode were selected. MS parameters, such as fragmentor energies and collision energy, were optimized. Quantification was performed using the MRM mode with transitions *m*/*z* 677.1/531.1 for ICA, 515.1/369.1 for ICA II, and 463.1/301.1 for IS (Figure 1).

To improve sensitivity in the positive ion mode of LC-MS/MS analysis, a small amount of 0.5% acetic acid was added into the mobile phase to help achieve high-quality resolution and the optimum peak shape in chromatograms. The optimum mobile phase conditions consisted of acetonitrile (A)–0.5% formic acid water (B) and gradient elution program was employed in the UPLC conditions: 0–1 min, 15% B; 1–2 min, 15%–40% B; 2–4 min, 40%–70% B; 4–5 min, 70%–90% B; 5–7 min, 90% B; 7–7.01 min, 90%–15% B; 7.01–8 min, 15% B.

### 2.2. Optimization of the Extraction Procedure

Precipitation of proteins using methanol or acetonitrile was tested as a method for stable plasma sample extraction. The amount of methanol or acetonitrile to be added was estimated 4–8 times. However, the desired extraction was not obtained through this method; furthermore, it was affected by the matrix effect and low recovery and did not exhibit good linearity for ICA and ICA II in plasma. Thus, a liquid–liquid extraction method using 10 times the acetic ether was employed for plasma sample preparation; this method exhibited stability and a high recovery rate of ICA, ICA II, and IS.

### 2.3. Method Validation

#### 2.3.1. Specificity and Selectivity

**Figure 2:**
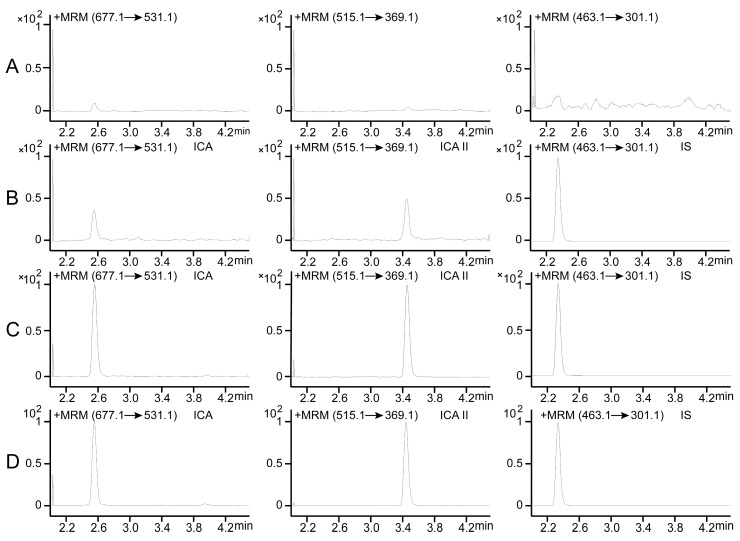
Representative MRM chromatograms of ICA, ICA II and IS in rat plasma. (**A**) A blank plasma sample; (**B**) A blank plasma sample spiked with ICA, ICA II at LLOQ and IS (10 ng/mL); (**C**) A blank plasma sample spiked with ICA, ICA II at 5.16 ng/mL and IS (10 ng/mL); (**D**) Plasma sample from a rat 60 min after oral administration of ICA at a dose of 30 mg/kg.

The typical chromatograms of ICA, ICA II and IS are shown in Figure 2A–D. Figure 2A–C show the representative ion chromatograms of blank plasma samples, lower limit of quantification (LLOQ) samples, and QC samples in the MRM mode, respectively. 

In addition, the ion chromatogram of a rat plasma sample captured 60 min after the oral administration of 30 mg/kg ICA is shown in Figure 2D. Under optimal method conditions, no endogenous peaks were observed at the retention time of ICA (2.55 min), ICA II (3.44 min), and IS (2.32 min) in the blank rat plasma, indicating no considerable endogenous interference in the MRM mode for ICA, ICA II, and IS during quantification.

#### 2.3.2. Calibration and LLOQ

Calibration curves for ICA and ICA II in rat plasma are linear and ranged from 1.03 to 1032 ng/mL with a correlation coefficient (r) of >0.997; the curves exhibited excellent linearity. Typical equations for the calibration curves were Y = 0.01019x − 8.731 × 10^−4^ (r = 0.9983 for ICA) and Y = 0.02204x − 31.24 × 10^−4^ (r = 0.9977 for ICA II). The mean LLOQ of analytes was estimated at the concentration of 1.03 ng/mL for both of ICA and ICA II. The present method is adequately sensitive for conducting additional pharmacokinetic studies.

#### 2.3.3. Assay Precision and Accuracy

Intra-day and inter-day precision and accuracy data for ICA and ICA II from QC samples are summarized in Table 1. The intra-day precisions of ICA and ICA II ranged from 0.4% to 3.2% and 0.5% to 4.8% at all QC levels, respectively. The inter-day precisions was within 3.0% for ICA and 6.2% for ICA II. For accuracy, relative errors were ranged from −3.0% to 4.8%. These results demonstrate that the developed method has satisfactory accuracy, precision, and reproducibility for quantifying ICA and ICA II in plasma samples.

**Table 1:** Intra- and inter-day precision and accuracy of the assay.

**Intra-Day (*n* = 6)**						
**Added (ng/mL)**	**Founded (ng/mL)**		**RSD (%)**		**Accuracy (RE %)**	
	**ICA**	**ICA II**	**ICA**	**ICA II**	**ICA**	**ICA II**
5.16	5.00 ± 0.16	5.27 ± 0.26	3.2	4.8	−3.0	2.1
103.2	106.4 ± 0.9	101.4 ± 4.8	0.8	4.7	3.1	−1.8
516.0	520.6 ± 2.0	521.9 ± 2.8	0.4	0.5	0.9	1.1
1032	1082 ± 12	1052 ± 17	1.1	1.6	4.8	2.0
**Inter-day (*n* = 6 Series per day, 6 days)**						
**Added (ng/mL)**	**Founded (ng/mL)**		**RSD (%)**		**Accuracy (RE %)**	
	**ICA**	**ICA II**	**ICA**	**ICA II**	**ICA**	**ICA II**
5.16	5.02 ± 0.15	5.31 ± 0.33	3.0	6.2	−2.8	3.0
103.2	106.9 ± 2.9	102.1 ± 5.9	2.7	5.8	3.6	−1.1
516.0	513.4 ± 6.0	518.6 ± 12.8	1.2	2.5	−0.5	0.5
1032	1052 ± 24	1031 ± 48	2.3	4.7	2.0	−0.1

#### 2.3.4. Recovery and Matrix Effects

Table 2 and Table 3 summarize the matrix effect and recovery of all analytes at different concentrations, respectively. The mean matrix effects ranged from 112.4% to 114.3% for ICA (RSD < 5%), from 91.6% to 104.9% for ICA II (RSD < 4.0%), and 105.0% for IS. The results indicated that no co-eluting substances significantly influenced the ionization of ICA, ICA II, and IS.

**Table 2:** Matrix effects for the detection of ICA and ICA II (*n* = 6).

Concentration (ng/mL)	ME (%)		RSD (%)	
	ICA	ICA II	ICA	ICA II
5.16	114.1	91.6	4.0	2.7
103.2	112.6	99.0	3.4	1.3
516.0	114.3	101.7	3.2	3.7
1032	112.4	104.9	3.1	3.7

**Table 3:** Recoveries for ICA and ICA II (*n* = 6).

Concentration (ng/mL)	RE (%)		RSD (%)	
	ICA	ICA II	ICA	ICA II
5.16	86.2	89.7	14.2	14.0
103.2	88.4	99.0	3.3	9.6
516.0	97.5	101.7	2.4	6.9
1032	101.8	105.8	3.2	7.7

Recovery was calculated using the following formula: RE (%) = spiked plasma/plasma extract × 100%. The mean extraction recovery of ICA and ICA II was >86.2% and that of IS (10 ng/mL) was 94.5%, indicating that the liquid-liquid extraction method extracted most of the analytes from the plasma samples with consistent, precise, and reproducible results.

#### 2.3.5. Stability of ICA and ICA II

The stability of ICA and ICA II was investigated using four parameters: short-term stability, freeze-thaw stability, post-preparation stability, and long-term stability. The results were assessed as relative error percentages (RE %) and compared with those of freshly prepared samples of corresponding concentrations. Accuracy was calculated using as (RE %) = stability samples/spiked plasma × 100%. RSD % ranged from 1.4% to 8.7%. These results are summarized in Table 4. All data were in the acceptable range, indicating that ICA and ICA II were stable under routine laboratory conditions and that no additional procedure was necessary to stabilize the compound for pharmacokinetic studies.

**Table 4:** Stability of ICA and ICA II.

Compound	Concentration (ng/mL)	Accuracy (RE %)			
		Post-Preparative 4 °C, for 24 h	Short-Term 25 °C, for 24 h	Freezing-Thawing −20 °C, 3 cycles	Long-Term −20 °C, 2 month
ICA	5.16	93.8	106.6	95.2	89.3
	103.2	104.2	111.6	106.8	105.5
	516.0	97.5	103.7	101.5	101.0
	1032	104.1	102.2	108.6	96.8
ICA II	5.16	98.2	102.8	93.9	84.0
	103.2	106.1	95.4	105.4	93.7
	516.0	98.7	92.0	96.6	89.7
	1032	103.1	87.0	100.5	88.4

### 2.4. Pharmacokinetic Study

The LC-MS/MS method has been successfully used in pharmacokinetic studies in rat plasma after ICA or ICA II administration. ICA and ICA II concentrations could be detected in all rat plasma samples for up to 12 h after administration. Table 5 and Table 6 list the main pharmacokinetic parameters of ICA and ICA II; these values are not in line with those reported in the literature [26,27,28]. This dissimilarity is due to rats not being administered pure ICA but EF or its preparation in most pharmacokinetic studies [26]. Only one study reported the administration of pure ICA to rats; however, the reported *C*_max_ and AUC_0–∞_ (ng × min/mL) for ICA and ICA II differ partly from those in our study [28]. Firstly, we predicted different solvent used to dissolve ICA in reference [28] and our study would affect the pharmacokinetic action of ICA. Because ICA is insoluble in water and ethanol, we used a mixed solution consisted of solutol HS 15, PEG 400 and water (15:15:70) as it would not induce hemolysis after intravenous administration. Moreover, Xu [28] used PEG 400 to dissolve ICA; however, PEG 400 cannot be used in an intravenous administration study because it may induce hemolysis. Therefore, we compared the pharmacokinetic properties of ICA dissolved in these two solvents after oral administration. The mean plasma concentration–time profiles of ICA are plotted in Figure S1. A pharmacokinetic analysis of ICA (Table S1) showed that all major pharmacokinetic parameters, including the *T*_max_, *C*_max_, MRT_0–t_, and AUC_0__-t_, of ICA dissolved in the mixed HS 15 solution were nonsignificantly different from those of ICA dissolved in PEG 400, indicating that different solvents have minimal effects in the pharmacokinetic properties of ICA. On the basis of the hypothesis that intestinal flora are responsible for the intestinal absorption and metabolism of Epimedium flavonoids [29], we suggest that the amount of intestinal flora and activity of enzymes in rats from different sources used in Xu’s and our study affect the intestinal absorption and hydrolysis of ICA. These findings warrant further research for exploring whether the absorption and hydrolysis of ICA vary among rats from different sources or among animal models with various diseases.

**Table 5:** Pharmacokinetic parameters of ICA and ICA II following a single oral dose of 30 mg/kg (Mean ± SD, *n* = 5).

Parameters	Oral Administration of ICA		Oral Administration of ICA II
	ICA	ICA II	ICA II
*T*_max_ (min)	15.0 ± 0.0	147.0 ± 17.1	24.0 ± 13.4
*C*_max_ (ng/mL)	27.2 ± 5.4	29.6 ± 6.8	103.6 ± 29.3
t_1/2z_ (min)	73.9 ± 107.4	261.7 ± 185.9	135.0 ± 127.4
CL (L/min/kg)	46.2 ± 6.0	4.98 ± 1.76	3.68 ± 1.02
MRT_0-t_ (min)	67.9 ± 54.0	271.3 ± 43.4	153.1 ± 68.6
MRT_0-∞_ (min)	83.1 ± 60.8	330.5 ± 107.1	189.4 ± 99.8
AUC_0-t_ (ng/mL × min)	642.7 ± 83.2	6403 ± 2146	8387 ± 2539
AUC_0-∞_ (ng/mL × min)	657.8 ± 80.4	6601 ± 2067	8711 ± 2579

**Table 6:** Pharmacokinetic parameters of ICA and ICA II following intravenous administration at 30 mg/kg, respectively ((Mean ± SD, *n* = 5).

Parameters	Intravenous Injection of ICA		Intravenous Injection of ICA II
	ICA	ICA II	ICA II
*T*_max_ (min)	5.0 ± 0.0	5.0 ± 0.0	5.0 ± 0.0
*C*_max_ (ng/mL)	3.543 × 10^4.^ ± 1.026 × 10^4^	79.8 ± 22.4	4292 ± 1364
T_1/2z_ (min)	319.8 ± 339.0	118.1 ± 57.3	281.9 ± 322.0
MRT_0-t_ (min)	27.4 ± 5.9	87.3 ± 47.8	98.4 ± 42.1
MRT_0-∞_ (min)	33.0 ± 14.3	91.5 ± 47.8	230.8 ± 314.3
AUC_0-t_ (ng/mL × min)	6.942 × 10^5^ ± 1.700 × 10^5^	2997 ± 1000	1.962 × 10^5^ ± 6.328 × 10^5^
AUC_0-∞_ (ng/mL × min)	6.954 × 10^5^ ± 1.687 × 10^5^	3013 ± 1002	2.111 × 10^5^ ± 4.906 × 10^4^

**Figure 3:**
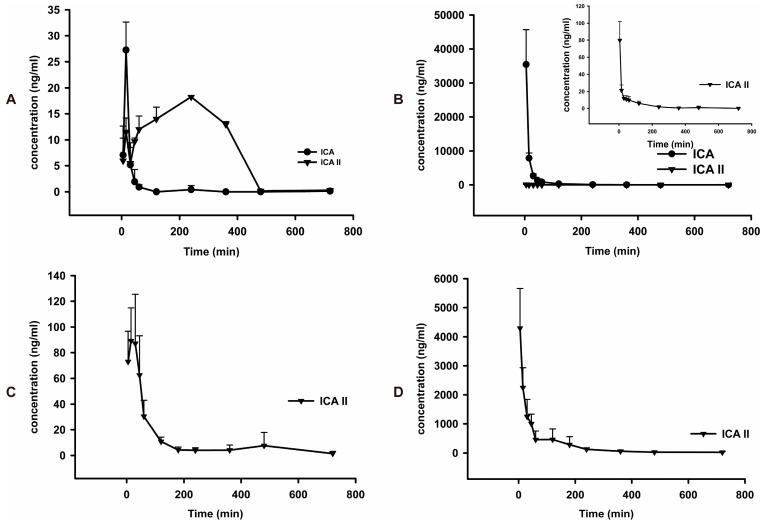
Mean plasma concentration-time curves of ICA and ICA II in normal rats (*n* = 5). (**A**) Intragastric administration of ICA at a dose of 30 mg/kg; (**B**) Intravenous injection of ICA at a dose of 30 mg/kg; (**C**) Intragastric administration of ICA II at a dose of 30 mg/kg; (**D**) Intravenous injection of ICA II at a dose of 30 mg/kg.

The mean plasma concentration-time profiles of ICA and ICA II after oral and intravenous administration of pure ICA and ICA II are shown in Figure 3. A pharmacokinetic analysis of ICA (Table 5) shows orally administrated ICA is rapidly absorbed in rats and is efficiently metabolized into its major metabolite, ICA II, which was detected in plasma at 5 min postdose. The time to reach the *T*_max_ of ICA was 15.0 ± 0.0 min. The mean AUC_0-t_ of ICA II (6403 ± 2146) was significantly higher than that of its parent drug, ICA (642.7 ± 83.2).After oral administration, 91.2% of ICA was transformed into ICA II. The MRT_0-t_ (min) of ICA II was longer than that of ICA. By comparing the pharmacokinetic parameters of ICA and ICA II (Table 6), we found that the *C*_max_ of ICA and ICA II was higher in rats administered ICA intravenously than in rats administered ICA orally; however, the AUC_0–t_ of ICA II (2997 ± 1000) was significantly lower in rats administered ICA intravenously than that in rats administered ICA orally (6403 ± 2146). Only 0.4% ICA was transformed into ICA II after intravenous administration. The main form of ICA in blood after oral administration was ICA II, whereas ICA remains unchanged in blood after intravenous administration. Thus, these findings suggest that the phaemacodynamic actions of ICA differ depending on the mode of administration. However, additional studies are required to verify these results. By individually comparing the pharmacokinetic parameters of ICA and ICA II after oral administration, we observed that the *C*_max_ and AUC_0–t_ of ICA II were 3.8 and 13 times higher, respectively, than those of ICA, indicating that ICA II is absorbed faster than ICA and metabolized slower than ICA. Therefore, the *in vivo* hydrolysis of ICA into ICA II facilitates ICA absorption. This result was consistent with that of the intestinal permeability test of ICA and ICA II [30]. A similar trend was observed in MRT_0–t_ in ICA and ICA II in rats. MRT_0-t_ of ICA II was almost twice that of ICA. Moreover, the absolute bioavailability of ICA II is approximately 4.1% ± 1.4%, which is significantly higher than that of ICA (0.1% ± 0.0%). This result indicates that ICA II has a higher systemic exposure than ICA does, which may be used to elucidate the differential effects of ICA and ICA II on the same disease.

The pharmacokinetic parameters of ICA II after intravenous administration was compared with those of ICA. After intravenous administration, the *C*_max_ and AUC_0–t_ of ICA were significantly higher than those of ICA II; these results differ from those obtained in rats after oral administration, suggesting that the site and pathway that metabolize ICA and ICA II in rats are completely different. ICA is a dual glucoside and is considered to be metabolized to ICA II after the removal of 7-*O*-glucose; subsequently, ICA II is metabolized to produce ICT after the further removal of 3-*O*-rhamnose. However, the microbial flora in the colon is apparently crucial for metabolizing ICA. After intravenous administration, ICA cannot be hydrolyzed without the aid of microbial flora. The *C*_max_ and AUC_0–t_ of ICA II in rats after intravenous administration were approximately 12.1% and 4.2% of those of ICA in rats after intravenous administration. ICA II appears to be metabolized faster than ICA is after intravenous administration. We hypothesized that ICA II metabolizes into other forms after intravenous administration. Thus, an LC-MS/MS method for quantifying ICA, ICA II, and ICT simultaneously in rat plasma was developed. However, only ICA II, as a metabolite of ICA, was detected in rat plasma after administering ICA and ICA II both orally and intravenously. ICT appears to not be produced during ICA II metabolism. Studies have shown that ICT is an intermediate product of ICA II, which is transformed into its sulfated and glucuronidated conjugated form in the liver [31]. Moreover, we found sulfated and glucuronidated conjugates of ICT in rat plasma after intravenous administration, indicating that ICA II in rats can be metabolized without the aid of microbial flora in the colon. Sulfated and glucuronidated conjugates of ICT can be quantified using the hydrolysis method with β-glucuronidase and sulfatase [32]; we will present this research in a future paper.

## 3. Experimental Section

### 3.1. Materials and Reagents

Purified ICA (purity > 95%), icaritin (purity > 98%) and diosmetin-7-*O*-β-d-glucopyranoside (IS) (purity > 98%), were purchased from Shanghai Yuanye Bio-Technology Co., Ltd. (Shanghai, China). ICA II was prepared by our laboratory and its structure was confirmed by comparison of its UV, IR, ESI-MS, ^1^H-NMR and ^13^C-NMR spectra (purity > 98%). Ethyl acetate was obtained from Sinopharm Chemical Reagent Co., Ltd. (Shanghai, China). HPLC-grade acetonitrile (ACN) was from Merck (Darmstadt, Germany). MS-grade formic acid was acquired from Anpel Scientific Instrument Co., Ltd. (Shanghai, China). Ultra-pure water was prepared with Milli-Q Ultrapure water purification system (Millipore, Bedford, MA, USA) and used for all analyses. All other reagents and chemicals were of analytical grade.

### 3.2. Apparatus and Analytical Condition

The samples were analyzed by an ultra-performance liquid chromatography-tandem mass spectroscopy (UPLC-MS/MS) (Agilent Corporation, Santa Clara, CA, USA), equipped with a binary pump (G4220A), an autosampler (G4226A) and a thermostated column compartment (G1316C). The Zorbax Eclipse plus C_18_ column (1.8 μm, 2.1 mm × 50 mm, Agilent Corporation) was performed for separation. 0.05% formic acid water (solvent A) and ACN (solvent B) were chosen to be the mobile phase. The following was the linear gradient elution program: 0–1 min, 15% B; 1–2 min 15%–40% B; 2–4 min 40%–70% B; 4–5 min 70%–90% B; 5–7 min 90% B; 7–7.01 min 90%–15% B; 7.01–8 min 15% B. The flow rate was set at 0.4 mL/min, and the injection volume was 5 μL. The optimized operating parameters of the ESI interface were set in positive mode as below: capillary voltage at 4000 V, the pressure of nebulizer gas (nitrogen) at 35 psi with a source temperature of 100 °C, gas temperature 350 °C with flow at 10 L/min. Multiple reaction monitoring (MRM), monitored the precursor-to-product ion transitions of *m*/*z* 677.1→531.1 for ICA, 515.1→369.1 for ICA II, and 463.1→301.1 for IS.

### 3.3. Preparation of Standard and Quality Control (QC) Samples

The standard stock solutions of ICA and ICA II were prepared by dissolving the requisite amount of ICA and ICA II in methanol. A series of standard working solutions at concentrations of 1.03, 2.06, 5.16, 10.32, 516.0, 103.2, 206.4, 516.0 and 1032 ng/mL both for ICA and ICA II were obtained by further dilution of the standard stock solution with methanol. The IS stock solution was prepared in methanol at 5.000 μg/mL and diluted with ethyl acetate to produce a working solution at a concentration of 10.00 ng/mL. All solutions were stored at 4 °C and taken to room temperature (20 °C) before use.

Blank rat plasma was collected from rats to prepare calibration and quality control (QC) samples, which were prepared by spiking the standard working solutions and IS working solution with blank plasma for method validation and pharmacokinetic studies. Calibration samples were made at the concentrations of 1.03, 2.06, 5.16, 10.32, 516.0, 103.2, 206.4, 516.0 and 1032 ng/mL both for ICA and ICA II with IS at the concentration of 10.00 ng/mL. QC samples were prepared at the concentrations of 5.16, 103.2, 516.0, and 1032 ng/mL for ICA and ICA II with IS at the concentration of 10.00 ng/mL. The spiked plasma samples at all the levels were stored at −20 °C for validation and subject sample analysis.

### 3.4. Sample Preparation

Plasma sample (50 μL) spiked with 500 μL acetic ether containing the IS was vortex-mixed for 5 min to extract the analytes and centrifugated at 15,000×*g* for 5 min at 4 °C. After solvent-solvent partition, the organic layer was clearly transferred and evaporated to dryness at 40 °C under a gentle stream of nitrogen. The residue was then reconstituted in 50 μL of methanol and vortex-mixed for 5 min. After centrifugation again at 15,000× *g* for 5 min at 4 °C, 5 μL of the supernatant was injected into the UPLC-MS/MS system for analysis.

### 3.5. Assay Validation

#### 3.5.1. Specificity and Selectivity

The selectivity of the method was investigated by comparing chromatograms of six blank plasma samples of different sources, plasma samples spiked with ICA, ICA II and IS, and a plasma sample after oral administration of ICA. Chromatograms were examined to determine the presence of any endogenous constituents which might potentially interfere with the analysis of ICA, ICA II and IS.

#### 3.5.2. Linearity and Lower Limit of Quantification (LLOQ)

The linear relationship of the method was evaluated by preparing nine different non-zero concentrations of plasma samples with ICA and ICA II using the extraction procedure mentioned in Section 3.4. The calibration curves were established by plotting peak area ratios of ICA or ICA II to IS vs the concentrations of ICA or ICA II. To evaluate linearity, plasma calibration curves were prepared and determined in six duplicate over the range 1.03–1032 ng/mL for ICA and ICA II with the same concentration (10.00 ng/mL) for IS. The calibration curve should meet the following criteria: deviations not more than 20% around LLOQ and not more than 15% above LLOQ. The contents of ICA and ICA II in the test samples were calculated using the regression parameters obtained from the standard curve. The LLOQ was defined as the concentration giving a signal-to-noise ratio of at least 10.

#### 3.5.3. Precision and Accuracy

The intra- and inter-day precision and accuracy were investigated by the determination of QC samples (six replicates for each concentration level) over six consecutive days. The concentrations were calculated using calibration curves obtained daily. The precision of the method at each QC concentration was expressed as the relative standard deviation (RSD) and the accuracy was described as relative error (RE), *i.e.*, (determined concentration − nominal concentration)/(nominal concentration) × 100%. The suitability of the precision and accuracy was assessed by the following criteria: RSD < 15% and RE < ±15%.

#### 3.5.4. Recovery and Matrix Effects

The recoveries of ICA, ICA II or IS in rat plasma (*n* = 6) after the extraction procedure were determined by comparing the peak areas of extracted ICA, ICA II or IS in plasma samples with that at the same concentration level dissolved in the processed blank sample (the final solution of blank plasma after extraction and dissolution with methanol). To evaluate the absolute matrix effect on the ionization of ICA, ICA II or IS, the peak areas of the compounds dissolved in the processed blank sample (the final solution of blank plasma after extraction and dissolution) were compared with those of the compounds dissolved in methanol. Four different QC concentration levels of ICA and ICA II with the same concentration of IS were evaluated (six replicates for each concentration level).

#### 3.5.5. Stability

QC samples (*n* = 6) of the analytes in plasma was prepared and analyzed to evaluate the stability for short-time stability with keeping QC sample for 24 h at room temperatures, freeze-thaw stability with three cycles of freezing-thawing at −20 °C, post-preparation stability by keeping the extracted QC samples in the autosampler at 4 °C for 24 h and long-term stability after storage of QC samples at −20 °C for 60 days. Concentrations following storage were compared with freshly prepared samples of the same concentrations.

### 3.6. Animal Protocol and Pharmacokinetic Study

Twenty male Sprague-Dawley rats, weighing 250–300 g, were supplied by the Laboratory Animal Center of the Shanghai University of Traditional Chinese Medicine. Animal welfare and experimental protocol were performed strictly in accordance with the Animal Care and Use Committee of Shanghai University of Traditional Chinese Medicine (Permit number: SCXK (Hu) 2012-0002). The rats were kept in an air-conditioned animal quarter at a temperature of 22–24 °C and relative humidity of 50% ± 10%, and had access to the standard laboratory food and water.

The rats were divided into four groups at random. Two groups were given a single dose of ICA solution at 30 mg/kg of body weight by intragastric or intravenous injection. Another two groups were given a single dose of ICA II solution at 30 mg/kg of body by intragastric or intravenous injection. ICA and ICA II were dissolved into the mixed solution consisted of Solutol HS 15, PEG 400 and water (15:15:70). Approximately 150 μL of blood samples were collected from the suborbital veniplex before dosing (0 min) and 5, 15, 30, 45, 60, 120, 240, 360, 480 and 720 min post-doing. The collected blood samples were transferred into heparinized tubes, and centrifuged at 4500 rpm for 10 min to separate out plasma. The plasma was then transferred to clean tubes and stored at −20 °C for preservation until analysis.

During routine analysis, each analytical run included six blank plasmas, a set of calibration samples, a set of QC samples and unknowns. Animal experiments were carried out in accordance with the local institutional guidelines for animal care of Shanghai University of Traditional Chinese Medicine.

### 3.7. Statistical Analysis

Pharmacokinetic data analysis was performed using DAS 2.1.1 software (Mathematical Pharmacology Professional Committee of China, Shanghai, China). Data are presented as Mean ± SD.

## 4. Conclusions

We have studied and compared the pharmacokinetic parameters of ICA and ICA II in rats. To the best of our knowledge, this is the first study analyzing and comparing the pharmacokinetic parameters of ICA and ICA II. This study clarifies the mechanisms underlying different pharmacokinetic actions of ICA and ICA II *in vivo*. First, we developed a simple and sensitive UPLC-MS/MS method for simultaneously quantifying ICA and ICA II; the time for detection was only 8 min, which is shorter than that required in other methods [27]. Moreover, the developed methods helps investigate the pharmacokinetic actions of ICA and ICA II. The main pharmacokinetic parameters of ICA and ICA II were studied in rats grouped according to the mode of administration (*i.e.*, oral or intravenous). The results showed that approximately 91.2% of ICA was transformed into ICA II after oral administration whereas only 0.4% of ICA was transformed after intravenous administration. The main form of ICA in blood after oral administration is ICA II, whereas ICA remains unchanged when administered intravenously. Individual comparisons of the pharmacokinetic parameters of ICA and ICA II after their administration revealed that ICA II is absorbed faster but metabolizes slower than ICA does after oral administration. By contrast, ICA II appears to metabolize faster into other forms, whereas ICA is not hydrolyzed into ICA II without the aid of microbial flora in the colon after intravenous administration. The results suggest that ICA and ICA II have distinct pharmacokinetic properties, and the insights gained facilitate future pharmacological action studies.

## Supplementary Materials

Supplementary materials can be accessed at: http://www.mdpi.com/1420-3049/20/12/19763/s1.
