# Supplementary material for: Comparative Pharmacokinetics Study of Icariin and Icariside II in Rats
Source: Molecules. 2015 Dec 1;20(12):21274–86. doi: 10.3390/molecules201219763 (PMC6332156; doi:10.3390/molecules201219763)
Supplement: Supplementary file 1 [file molecules-20-19763-s001.pdf]

Supplementary Materials: Comparative Pharmacokinetics Study of Icariin and Icariside II in Rats

Tao Cheng, Yong Zhang, Tong Zhang, Lu Lu, Yue Ding and Yuan Zhao

Table S1. Pharmacokinetic parameters of ICA after oral administration of ICA dissolved in different solvents at 30 mg/kg (Mean ± SD, n = 4).

Parameters	ICA Solution Prepared by PEG400	ICA Solution Prepared by HS 15 Mixed Solution
T_{max} (min)	30.1 ± 12.2	18.8 ± 7.5
C_{max} (ng/mL)	24.7 ± 15.4	20.1 ± 13.7
$t_{1/2z}$ (min)	152.7 ± 103.3	93.1 ± 22.1
CL (L/min/kg)	46.8 ± 16.9	43.2 ± 10.9
MRT_{0-t} (min)	75.9 ± 17.4	85.2 ± 14.6
$MRT_{0-\infty}$ (min)	109.1 ± 26.0	99.9 ± 22.1
AUC_{0-t} (ng/mL × min)	664.4 ± 214.2	699.4 ± 159.9
$AUC_{0-\infty}$ (ng/mL × min)	700.0 ± 220.7	724.6 ± 164.3

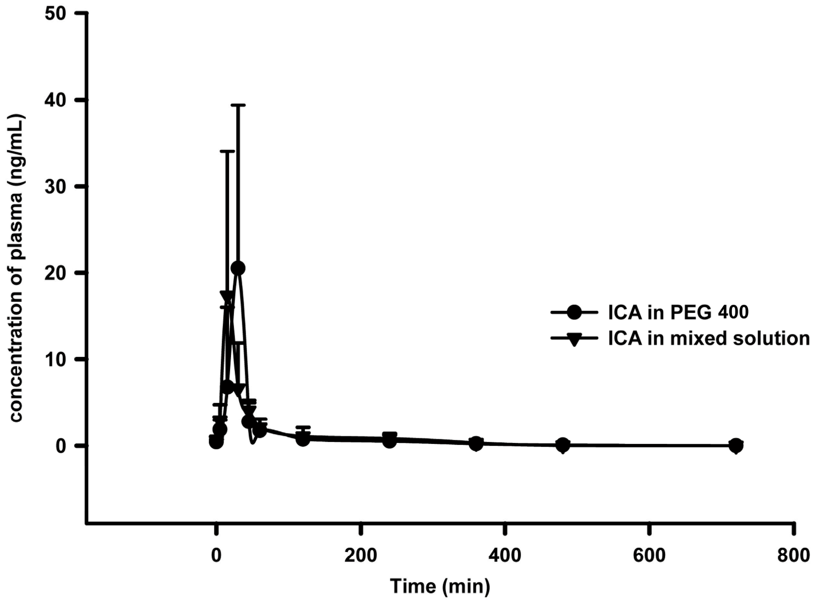

Figure S1. Mean plasma concentration-time curves of ICA in normal rats after oral administration of ICA dissolved in PEG400 and a mixed solution consisted of Solutol HS 15, PEG 400 and water (15:15:70) (n = 4).
